# Extrahepatic bile duct hepatocellular carcinoma due to recurrence of hematogenous metastasis 50 months after hepatectomy

**DOI:** 10.1186/s40792-017-0305-3

**Published:** 2017-02-16

**Authors:** Hiroyuki Kumata, Shigehito Miyagi, Keigo Murakami, Atsushi Fujio, Yasuyuki Hara, Chikashi Nakanishi, Naoki Kawagishi, Hironobu Sasano, Takashi Kamei, Noriaki Ouchi

**Affiliations:** 1 Division of Advanced Surgical Science and Technology, Graduate School of Medicine, Tohoku University, 1-1 Seiryou-machi, Aobaku, Sendai, 980-8574 Japan; 2 Department of Pathology, Graduate School of Medicine, Tohoku University, Sendai, Japan

**Keywords:** Carcinoma, Hepatocellular, Neoplasm metastasis, Recurrence

## Abstract

**Background:**

Recurrent hepatocellular carcinoma (HCC) in the extrahepatic bile duct is rare with most cases diagnosed after manifesting sudden obstructive jaundice. Here, we report an extremely rare case of recurrent HCC in the common bile duct due to hematogenous metastasis.

**Case presentation:**

A 66-year-old man underwent an extended left hepatectomy for HCC in the medial segment of the liver. Fifty months later, he presented with sudden obstructive jaundice. Endoscopic retrograde cholangiography showed a space-occupying lesion in the common bile duct, which was suspected as cholangiocarcinoma. Therefore, he underwent extrahepatic bile duct resection and choledochojejunostomy with lymph node dissection. Macroscopically, a polypoid tumor and several nodular tumors were found in the common bile duct, which was obstructed by a tumor thrombus. Histopathologically, the tumors were diagnosed as metastases from the HCC resected 50 months before. Several distinct, nodular tumors were observed in the subepithelium of the common bile duct and had invaded some blood vessels. These findings support the conclusion that the HCC metastasized hematogenously to the extrahepatic bile duct.

**Conclusions:**

Recurrent HCC in the extrahepatic bile duct due to hematogenous metastasis is rare, and it is difficult to diagnose. Further similar cases should be accumulated for clarifying the pathological mechanism.

## Background

Curable hepatocellular carcinoma (HCC) is gradually increasing because of improved follow-up and imaging diagnosis protocols for chronic liver diseases. However, a therapeutic strategy for metastasis and recurrence of HCC remains a difficult topic. Recurrent HCC in the extrahepatic bile duct is somewhat rare, and almost all cases are diagnosed after the manifestation of sudden obstructive jaundice [1]. We report a case of recurrent HCC in the common bile duct due to hematogenous metastasis with tumor thrombus 50 months after primary hepatectomy.

## Case presentation

**Fig. 1:**
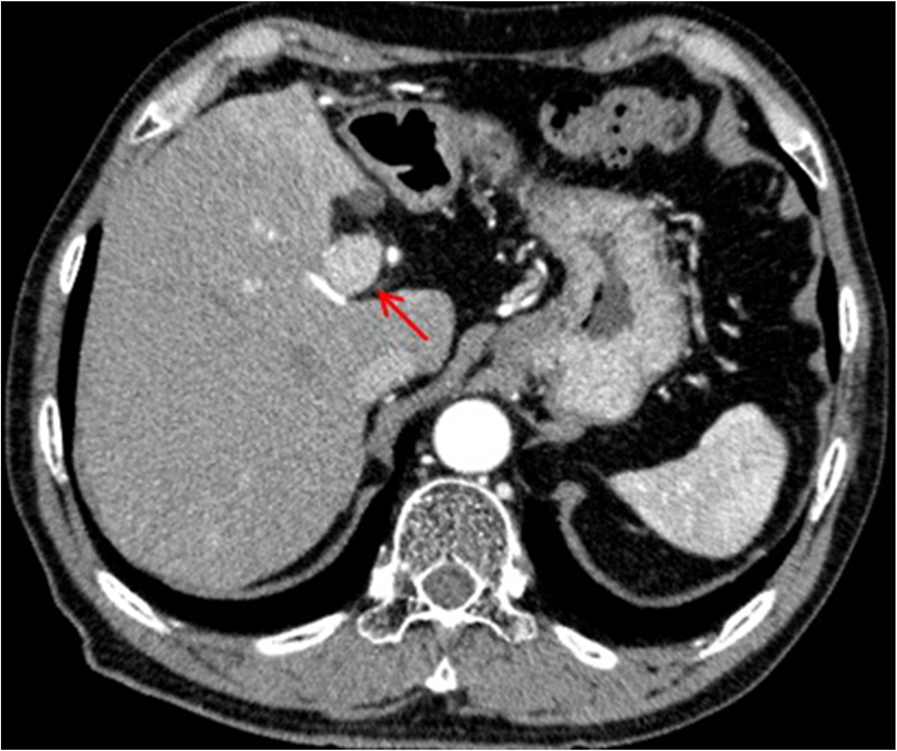
Computed tomography findings. There was a 30-mm contrast-enhanced space-occupying lesion in hepatic segment 4 at the early phase (*arrow*). The lesion presented with washout at the delay phase

**Fig. 2:**
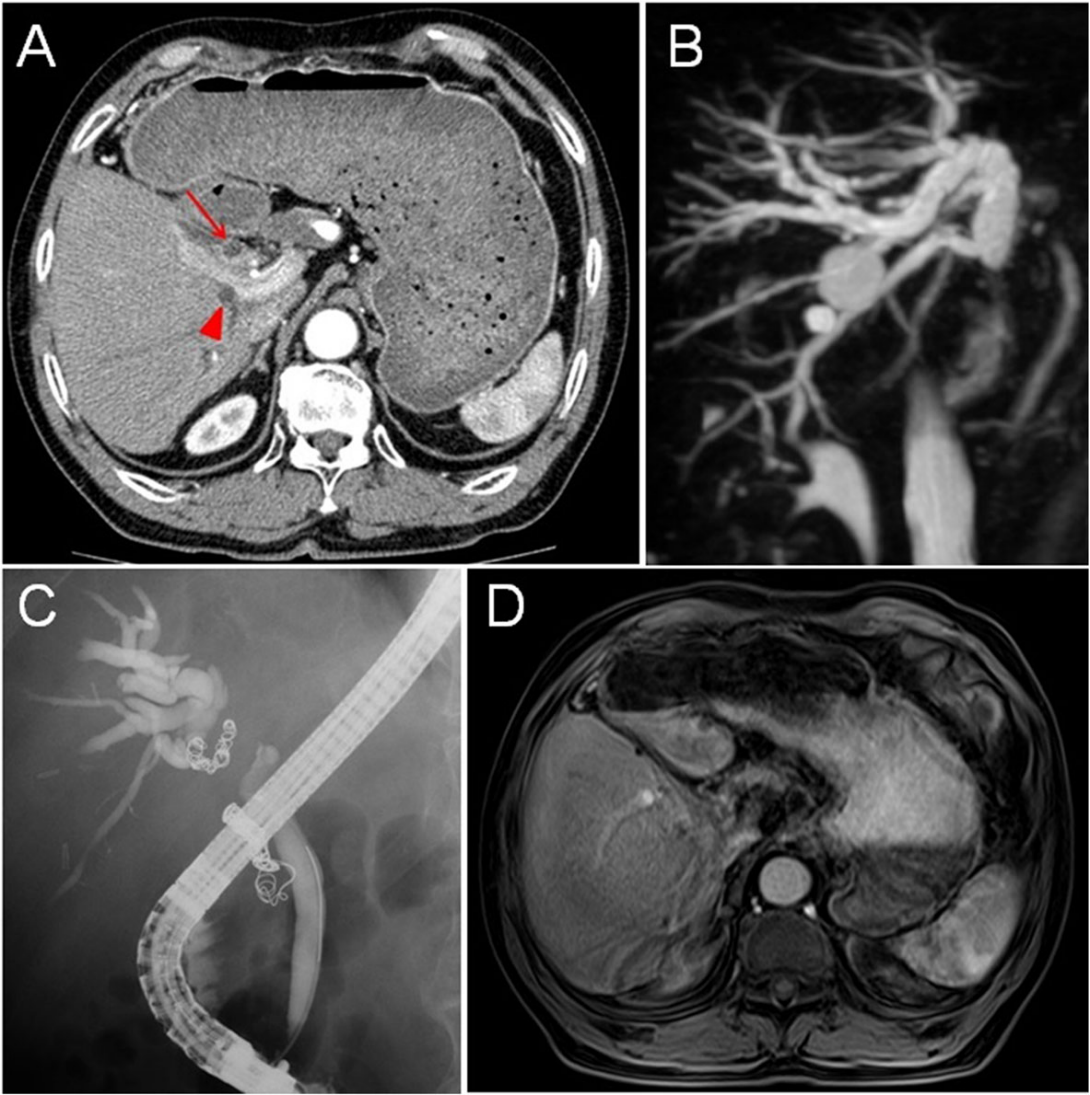
Preoperative imaging findings. **a** Computed tomography showed that cholangiectasis was observed in both anterior (*arrow*) and posterior (*triangle*) segments of the right hepatic lobe, but no definitive mass lesions were found in the liver. **b** Magnetic resonance cholangiopancreatography showed cholangiectasis from the intrahepatic bile ducts to the middle segment of the common bile duct. **c** Endoscopic retrograde cholangiopancreatography showed that the common bile duct was obstructed by a tumorous lesion covering the upper and middle segment of the common bile duct. **d** Dynamic liver magnetic resonance imaging showed that the tumor became ambiguous due to the influence of the biliary drainage, and it had poor contrast effect in all contrast phases (the displayed image is early phase image). No definitive mass lesions except for simple cysts were found in the liver

A 66-year-old man with hepatitis C underwent an extended left hepatectomy for HCC in the medial segment of the liver. Preoperative dynamic computed tomography (CT) and intraoperative evaluation revealed no lymph node metastasis (Fig. 1). Histopathologically, the 3.9-cm-diameter tumor was confirmed to be moderately differentiated HCC with trabecular structure. In addition to microscopic venous invasion, tumor cells had extensively invaded the portal vein, resulting in a tumor thrombus in the left portal vein. There were several daughter nodules of intrahepatic metastasis around the main tumor. Although the resected margin was tumor negative, tumor cells had invaded the serosa of the liver. There was no obvious hepatic artery or bile duct invasion. His postoperative course was good, and his alpha fetoprotein (AFP) and des-gamma-carboxy prothrombin (DCP) levels were within normal limits. Follow-up CT examinations were performed every 6 months. Fifty months after the hepatectomy, he was referred to our hospital with sudden obstructive jaundice. Laboratory studies revealed hyperbilirubinemia (total bilirubin 10.5 mg/dl) and a slight increase of DCP (85 mAU/ml); AFP levels were normal. CT revealed dilation of the intrahepatic biliary tree, but no definitive mass lesions were found in the liver (Fig. 2a). In addition, there was an aneurysm in the common hepatic artery. Magnetic resonance cholangiopancreatography revealed cholangiectasis from the intrahepatic bile ducts to the middle segment of the common bile duct (Fig. 2b). Wall thickening and a mass showed low signal by T2-weighted imaging and high signal by diffusion-weighted imaging in the middle segment of the common bile duct. Similar to CT, no definitive, hepatic mass lesions were found. Fluorodeoxyglucose positron emission tomography also showed solitary uptake in the middle segment of the common bile duct. Endoscopic retrograde cholangiopancreatography (ERCP) showed that the common bile duct was obstructed by a tumorous lesion covering the upper and mainly the middle segment of the common bile duct (Fig. 2c). Following lesion biopsy, external biliary drainage was performed using an endoscopic nasobiliary drainage (ENBD) tube. After placing the ENBD tube, the patient underwent dynamic liver magnetic resonance imaging. In the dynamic study, the tumor became ambiguous due to the influence of the biliary drainage, and it had poor contrast effect in any contrast phase (Fig. 2d). Cytological examination of the biopsy specimen revealed atypical cells forming papillary arrangement, which was suggestive of cholangiocarcinoma rather than HCC. However, positivity was observed not only for AE1/AE3 but also for Hep par 1. Although it was unclear whether the tumor was cholangiocarcinoma or HCC from these pathological features, the imaging studies suggested that the tumor seemed to develop from the bile duct wall, which implied cholangiocarcinoma rather than HCC. The patient was to undergo surgical resection for diagnostic treatment. Upon selection of the surgical procedure, we considered the pathological findings, patient’s hepatic spare ability, local tumor extent based on ERCP, and common hepatic artery aneurysm that posed a high risk for pancreaticoduodenectomy. Therefore, resection of extrahepatic bile duct and choledochojejunostomy with lymph node dissection were performed on suspicion of solitary cholangiocarcinoma. Complete resection (R0) was achieved by pathologically determining negative surgical margin during surgery.

**Fig. 3:**
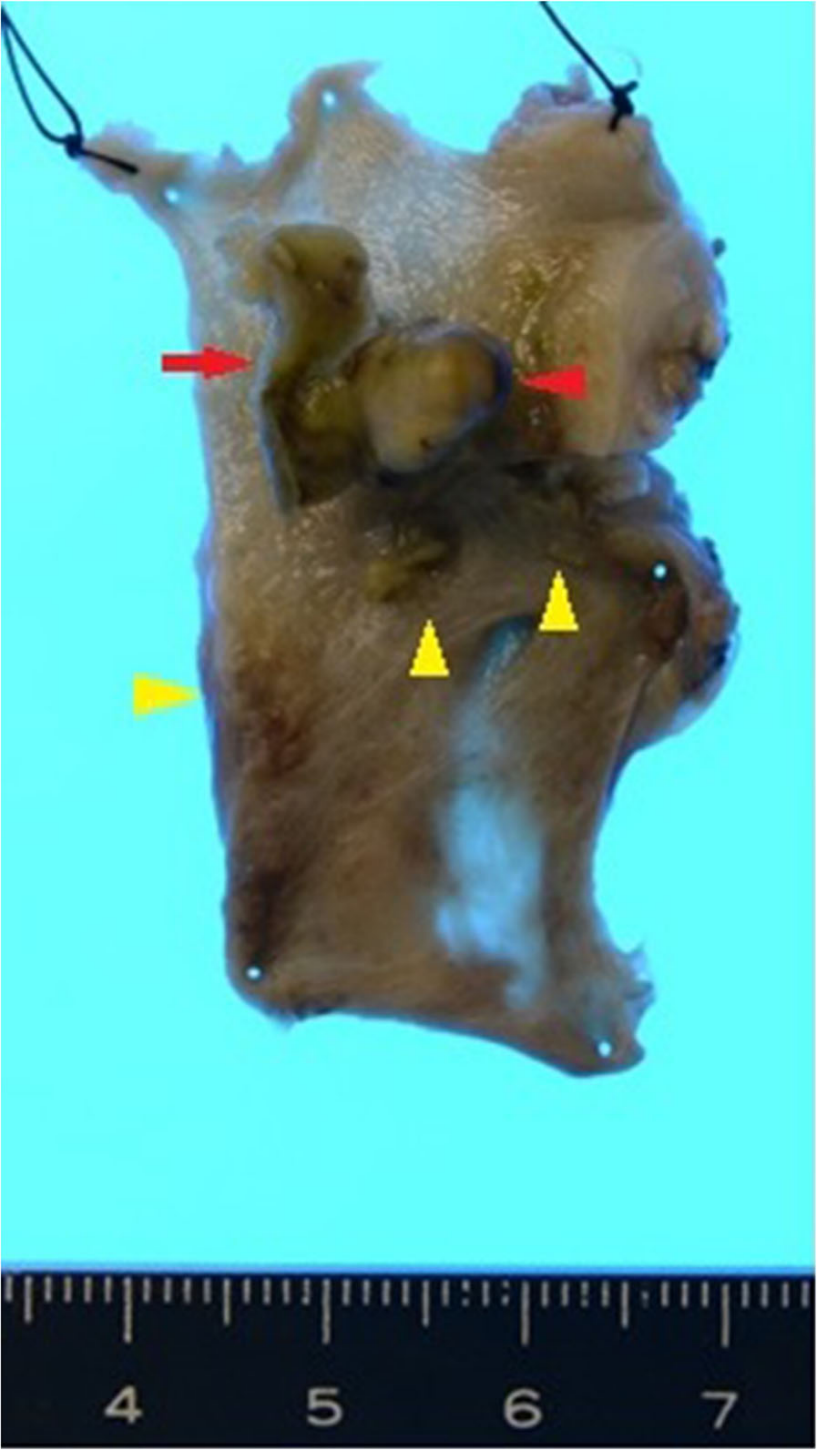
Macroscopic findings. A polypoid tumor (*red triangle*) of approximately 10-mm diameter continuous with the epithelium and a tumor thrombus (*arrow*) in the dilated common bile duct. Several subepithelial nodules were observed (*yellow triangles*)

**Fig. 4:**
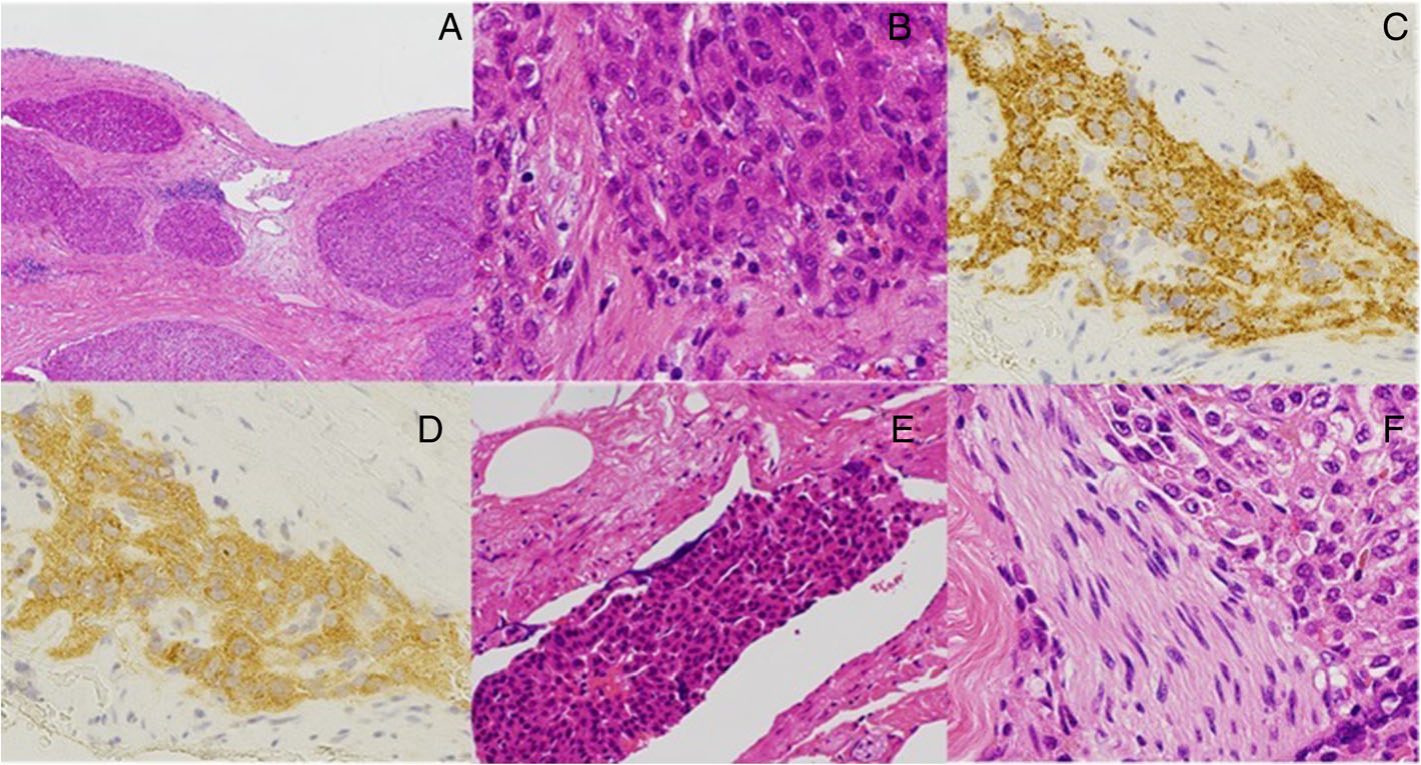
Microscopic findings. **a** Distinct, numerous nodular tumors covered by epithelium that did not expose the bile duct lumen (hematoxylin/eosin (HE)). **b** These lesions were diagnosed as moderately differentiated HCC (HE). HCC immunostaining for **c** Hep par 1 and **d** Glypican 3. The nodular tumors showed **e** microscopic venous and **f** perineural invasion (HE)

Macroscopically, an approximately 10-mm-diameter polypoid tumor continuous to the epithelium and a tumor thrombus were found in the dilated common bile duct, and several nodular tumors were observed subepithelially (Fig. 3). The lesions and tumor thrombus were diagnosed as moderately differentiated HCC (Fig. 4a, b). Immunohistochemical staining revealed that they were positive for Hep par 1 (Fig. 4c) and Glypican 3 (Fig. 4d) but negative for both cytokeratin 7 and 20. Moreover, these tumors showed microscopic venous (Fig. 4e) and perineural invasion (Fig. 4f) analyzed using D2-40 and anti-CD31 antibodies. Microsections showed distinct, numerous nodular tumors that were covered by the epithelium and that did not invade the epithelium (Fig. 4a, b). However, the polypoid lesion was not completely covered by the epithelium, protruding toward the lumen of the bile duct with invasion. The tumor thrombus was disconnected to both the tumor and the epithelium. No metastasis was observed in dissected lymph nodes. On the basis of these findings, the patient was diagnosed with recurrent HCC in the extrahepatic bile duct due to hematogenous metastasis. Six months after the surgery, he developed multiple intrahepatic metastases and was still alive at the time of drafting this report.

## Discussion

HCC in the major bile duct, especially in the common bile duct, can cause obstructive jaundice [1]. Such HCCs are called icteric-type HCCs and tend to be complicated by advanced clinical stage [2]. The most typical clinical finding is obstructive jaundice confused with cholangiocarcinoma [1]. Although most cases seem to originate from tumor thrombi, the thrombi rarely stick to or invade the bile duct wall [3, 4]. This case is unique because the recurrent HCC tumor due to hematogenous metastasis penetrated the bile duct wall and generated a tumor thrombus 50 months after the initial surgery. To the best of our knowledge, except for our case, there has been only one report of hematogenous HCC recurrence in the bile duct [5]. However, their diagnosis was based only on circumstantial evidences, i.e., clinical and microscopic. Besides HCC, there are three cases of colon [6, 7], two of kidney [8, 9], and one of lung [10] cancers that reportedly showed metastasis to the common bile duct. All the cases were strongly considered that hematogenous metastasis from the viewpoint of the anatomical positional relationship between the common bile duct and each organ.

There are some rare reports of extrahepatic HCC without primary hepatic parenchymal lesions [2, 11]; hence, the bile duct tumors in this case might be new HCC lesions. However, the bile duct tumors in this case were considered recurrence because of the morphological similarities and venous invasion. Furthermore, most cases of primary extrahepatic HCC are continuous and localized masses [12], whereas the tumors in this case are extensively distinct and scattered.

HCC recurrence can be explained by the following points: (i) invasive recurrence from microscopic bile duct invasion; (ii) infiltrating recurrence of tumor thrombus having been incompletely removed during primary hepatectomy; (iii) invasion from the surrounding tissues of the common bile duct; or (iv) recurrence of hematogenous metastasis with microscopic venous invasion. In this case, no microscopic bile duct invasions were observed in the primary or recurrent HCCs. Furthermore, most recurrences originating from tumor thrombus have been reported to develop within a year of surgery [4], unlike this case in which recurrence occurred 50 months after primary hepatectomy. Although a polypoid tumor was observed to break through a part of the epithelium, numerous lesions were extensively observed subepithelially. Therefore, we consider that the recurrence originated from hematogenous metastasis with microscopic venous invasion, and the tumor thrombus was separated from the polypoid tumor.

In this case, because (a) there were severe portal invasion, (b) tumor thrombus was located in the left portal vein, and (c) there were several daughter nodules of microscopic intrahepatic metastasis, the patient had high chance of recurrence. Despite such high-risk histopathological findings, it is very interesting that he had a disease-free interval of 50 months before recurrence.

The effectiveness of surgery for recurrence originating from tumor thrombus has been previously reported [13, 14]. Although thrombectomy is effective in the treatment of bile duct tumor thrombus, positive outcomes of bile duct resection with thrombus have been reported when tumor thrombus has invaded the bile duct epithelium. However, the recurrence in this case originated from hematogenous metastasis and exhibited aggressive behavior after the bile duct resection. Therefore, further cases and studies are needed to reveal the effectiveness of surgery.

## Conclusions

We report a patient who developed an extremely rare recurrent HCC in the common bile duct due to hematogenous metastasis. Recurrent HCC in the extrahepatic bile duct is rare and presents a poor prognostic course. Further similar cases should be accumulated for clarifying the pathological mechanism.

## Abbreviations

AFP: Alpha fetoprotein
CT: Computed tomography
DCP: Des-gamma-carboxy prothrombin
ENBD: Endoscopic nasobiliary drainage
ERCP: Endoscopic retrograde cholangiopancreatography
HCC: Hepatocellular carcinoma
HE: Hematoxylin/eosin
